# The Analysis of the Artifacts due to the Simultaneous Use of Two Ultrasound Probes with Different/Similar Operating Frequencies

**DOI:** 10.1155/2013/890170

**Published:** 2013-03-31

**Authors:** Samreen Amir, B. S. Chowdhry, Manzoor Hashmani, Musarrat Hasan

**Affiliations:** ^1^Sir Syed University of Engineering & Technology, Karachi, Pakistan; ^2^Mehran University of Engineering & Technology, Jamshoro, Pakistan; ^3^Iqra University, Karachi, Pakistan; ^4^Institute of Ultrasound Imaging, P.O. Box 75300, Karachi, Pakistan

## Abstract

The ultrasound imaging has the potential to become a dominant technique for noninvasive therapies and least invasive surgeries. Few cases may require using multiple probes of different units with different modes of ultrasound on the same patient. It generates imaging artifacts, which makes it complicated to gather information from the acquired image. This study was to identify and analyse the artifacts which are produced by simultaneous use of two probes with different/same operating frequencies. Six imaging studies were performed. First of all, the imaging artifacts of the 3.5 MHz and 6 MHz center frequencies with similar (longitudinal) positions of the probes. Secondly, with similar operating frequencies the 6 MHz probe changed from longitudinal to transverse placement to analyse the resulting artifacts. The third study was done with transverse placement of 3.5 MHz probe. The rest of the three cases were just the repetition with common pulse frequencies. Such artifacts in 3D ultrasound images are more obscure than the other artifacts associated and reported.

## 1. Introduction

The ultrasound imaging has been very common in medical applications for the diagnosis and therapy purposes. Its primary use is to know the fetal well-being in the womb. There were several problems in acquiring better quality image due to artifacts associated with this technique [1]. Some are inherent, and few are due to external sources [2]. 

The artifact of the image is the attribute that is not present in the original image object. Sometimes, an artifact of the image is the result of a malfunction of the device used for imaging, sometimes the result of natural processes, or the properties of the human body like obesity and so forth. Ultrasonic artifacts may be understood as the reflection or echo, which appears on the screen and represents the real anatomical structure which is incorrect [1]. An artifact can be a bunch of the false values that might be introduced through the imaging system or by interaction of ultrasound with adjacent tissue of misleading information. The ultrasound artefacts can be classified as to their sources which are physiologic (for, e.g., motion, different speeds of sound, and acoustic impedance of tissues), equipment (dimension of the ultrasound beam and the converters array), and technical imaging (mode B, spectral Doppler, and color Doppler ultrasound) [2]. These artifacts may be reduced by several techniques like amplitude shadowing, apodization [3], frame averaging, Wall filters [4], and so forth.

Ultrasound transducers are made to vibrate through piezoelectric effect. The short bursts of these vibrations are transmitted into the tissue. With the speed of 1540 m/s they travel within soft tissues. The resulting transit time is usually 6.5 microsec/mm [5]. It is assumed that the velocity of ultrasound waves does not change in a round trip so the time taken for the wave to return to the transducer can be used to determine the depth of the object under examination. The transducer elements are normally serving as a transceiver. For a reflecting signal it converts the mechanical energy back into an electrical signal to form an image. The probe also emits some back reflections which are encountered by a sound absorbing substance. An acoustic lens is also deployed to focus the emanating sound waves. The received signal is then processed by software to convert the scan lines into a meaningful image to be displayed at a monitor.

Several multielement probes are now obtainable for dynamic focusing. In such probes, each transducer element acts as an individual transceiver and has its own circuit. The primary advantage is that the ultrasound beam can be methodical by changing the pulse triggering time of each element. The variety of transducer types is obtainable and can select the suitable one for best imaging. There are different shapes and sizes of probes. The field of view of the probe is determined by its shape. A linear probe contains one or more acoustic linear array transducer elements arranged in a line to send pulses of sound into a material. The near field and large footprint of the linear array make it different from convex probes [6]. Convex probes yield sector images. Convex transducers are today customary on almost every modern scanner [7]. There is no major difference in focusing and beam sweeping algorithms of a convex or curvilinear and linear array transducer, except for the shape of the probe and the format of the generated image. 

While conducting an experiment regarding fetal movements as a result of Pulsed Wave Doppler (PWD) ultrasound, [8] we encountered the severe artifacts in the acquired image. The protocol of that experiment includes two ultrasound units operating at different center frequencies: one for imaging and another for stimulus. The imager has to be kept blind about the application of the stimulus; that is the reason why we decided to go for two individual units of US. But what we observed is that image was badly affected by the application of PWD stimulus from the other unit. The use of two simultaneous probes may also be required for therapy plus imaging processes.

This leads us to design a new protocol for that study as well as another one to investigate and analyze these artifacts. The latter part of the paper shall explain the material and methods, results and discussion. 

## 2. Materials and Methods

**Figure 1:**
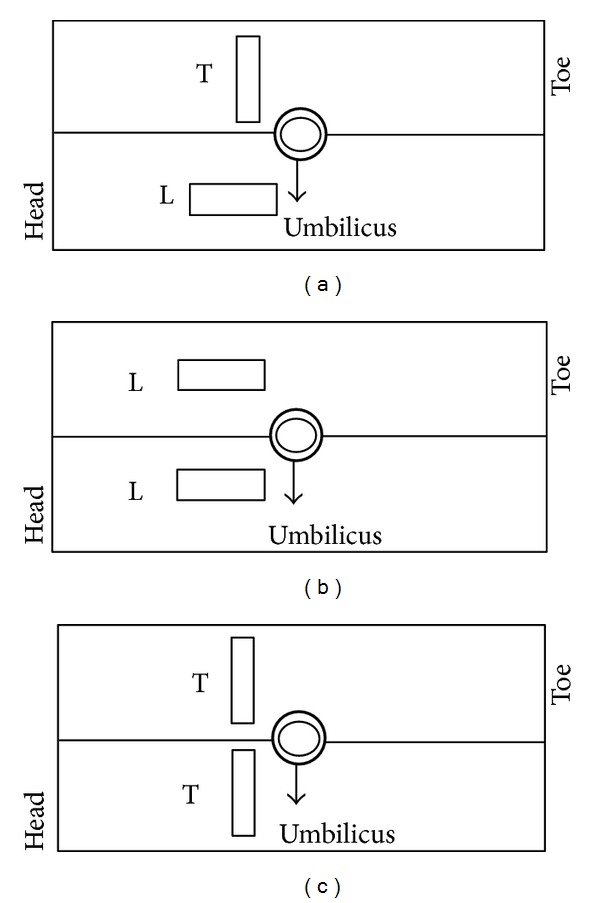
Illustration of orientation of ultrasound probes for the experiment.

The two machines used were Xario Toshiba SSA-660 Doppler Ultrasound and Medison SonoAce Pico Ultrasound Machine. The standard convex probes were used with an intensity level of 95 mW/mm^2^. The transducer operates in a broadband 3.5 MHz and 6 MHz and scans a 3D volume by electronically steering the acoustic beam in real time. The focal length and the imaging depth could be varied as desired. The 3D images were saved as DICOM (Digital Imaging and Communications in Medicine) files. A desired frame of the movie file was saved as a TIFF image.  Figure 1 shows the placement pattern of the probes over patient. The subject taken was an OB patient with low risk singleton fetus of gestational age of 28 weeks. The internal abdominal wall thickness index was less than 1 to have better quality of image which may reduce due to patient's obesity. The approval was obtained from the Institutional Review Board of Institute of Ultrasound Imaging, Karachi. The experiments were conducted at Dr. Musarrat's Ultrasound clinic under the supervision of Dr. Musarrat Hassan. The protocol was based on FDA approved equipment with operating powers under ALARA guidelines and purely noninvasive method. All the principles given in the declaration of Helsinki are followed.

The pattern applied on the patient was longitudinal and transversal which are designated as L and T, the configuration of which is shown in Table 1. Figures 1(a) and 1(b) represent the position of the probe in LT and LL direction, while Figure 1(c) shows the TT arrangement of the standard convex probe. Along with these combinations the analysis of the artifacts was performed which are discussed in the latter section.

## 3. Results and Discussion

**Figure 2:**
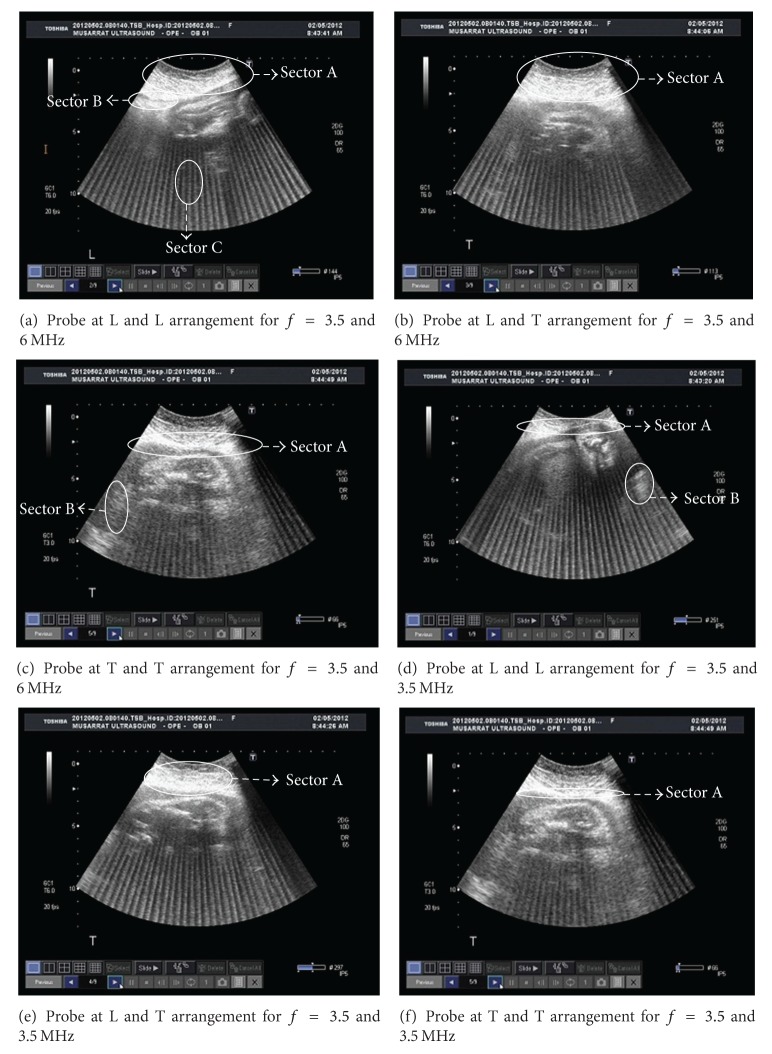
Artifacts images of all six cases.

**Figure 3:**
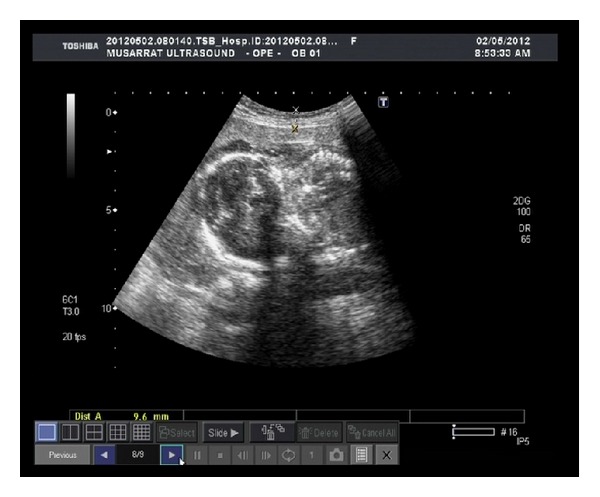
Original image for reference.

Most imaging artifacts produced are due to either reverberation or side lobes. Two types of reverberation artifacts and 2 types of side lobe artifacts were observed. These are described below along with an additional artifact. The comet tail artifact is shown in almost all the observed cases given in Figure 2.  Figure 3 is provided as a reference case. This image is acquired using one of the probes over the same patient. This image has no artifacts and hence is referred to as original image.  Figure 2(a), corresponds to the ultrasound image of case 1 of Table 1. It can be observed from sector A, that there is a bright area under the abdominal wall. It might be due to the multiple echoes generated that are formed and received by the transducer. These echoes are usually taken as the accumulation of the scattered signal while reflecting back. This figure has severe artifacts so that the fetus is not visible. The presence of finger can be seen in sector B which might be due to the presence of the guided wave artifact [9]. Comet tail artifact is marked as sector C in Figure 2(a). The several tails are formed as every element of probe 1 has a counter interferer in the form of probe 2 and vice versa. The images shown in the paper are taken from only one machine because monitors of both machines were having the identical image. This is the reason why we may say that effect of interference of probe 1 on probe 2 is same as of probe 2 on probe 1. Side lobe artifacts are ubiquitous. The strong, curved, and highly reflecting surfaces at near distance such as large cysts, the urinary bladder, or gallbladder constitute the specular side lobe artifact, while gases present close to such structures may give rise to the diffuse artifact. Hence the side lobe artifact is the byproduct of the hyperechoic structure present near the object under examination [10]. Since the reference image does not anticipate any of the aforementioned phenomenon, so the reason of having side lobe artifact in this particular scenario is fairly the energy particles of the US beam transmitted from the adjacent probe. It is evident that the fetal lower boundary is almost invisible in all observed images. It shows that this artifact reduces the lateral resolution of the image. It is also important to mention here that the types of artifacts not only are confined to reverberation and side lobes but could also be due to range ambiguity, in this particular case under study. A different pattern is observed in case 2 of Table 1, where the bright area at the top of the image has increased. The beams are perpendicular to each other in the image. The higher frequency transducer is placed at a right angle to the 3.5 MHz pulse. Since the higher frequency will have higher attenuation and less penetration as compared to low frequency [9], the reflecting signal would be a mixture of 6 MHz pulse and a delayed 3.5 MHz pulse. The reflections with delay less than that of 3.5 MHz signal will be received at both probes. The 6 MHz probe will process the signal successfully as compared to the 3.5 MHz probe. The apodization and delay lines of latter probe is set according to the 3.5 MHz signal which does not meet the values of weights required for the reception of 6 MHz signal by the digital filter. That could give rise to a hardware artifact which is defined in the Introduction Section. The image in case 3 given in Figure 2(c) has severe artifact with increased brightness at the bottom too. In the previous images the side lobe artifact has just appeared at the top of the image and blurred the fetal details, but in the current case the complete image is somehow affected by the side lobe artifact of both types. The comet tails are not even as sharp as they could be seen in Figures 2(a) and 2(b).

The rest of the three images to be discussed here are the cases of the same operational US center frequency. The same artifacts are still present in the images but with different attributes and proportions. A much better image can be observed in Figure 2(d). The fetal details are bit meaningful than the rest. Furthermore the overall brightness of the image is also reduced. Sector B shows the presence of curvilinear segments which is due to the specular side lobe artifact [9]. The image related to scenarios 5 and 6 of Table 1 shows strong artifacts due to which the images are not understandable forms except for the portion under sector A which shows the image due to the reflection on the bone.

## 4. Conclusion 

Two types of reverberation artifacts and 2 types of side lobe artifacts were observed. The artifacts were mainly comet tail, and guided wave reverberation artifact, and spectral and diffractive side lobe. Such artifacts in 3D ultrasound images are more obscure than the other artifacts associated and reported. The change in specified placement of probes and the difference in operating center frequency have no effect on the overall reduction of these artifacts. The placement has shown slight variation in the proportion of the artifact. It is observed that the arrangement of “LL” images is mostly affected with comet tail while “TT” images are affected by specular and diffuse artifacts. Certain other prearrangements of the probes with different angles may be tested in future studies to verify the effect. 

## Figures and Tables

**Table 1:** Combination of frequencies and probes' orientation.

Cases	Frequencies		Position	
Case 1–6	*F*1 (MHz)	*F*2 (MHz)	P1	P2
Case 1	3.5	6	L	L
Case 2	3.5	6	L	T
Case 3	3.5	6	T	T
Case 4	3.5	3.5	L	L
Case 5	3.5	3.5	L	T
Case 6	3.5	3.5	T	T
